# Long-Term Outcomes of Nonsurgical Treatment of Thumb Carpometacarpal Osteoarthritis: A Cohort Study

**DOI:** 10.2106/JBJS.22.01116

**Published:** 2023-10-30

**Authors:** Lisa M.J. Esteban Lopez, Lisa Hoogendam, Guus M. Vermeulen, Jonathan Tsehaie, Harm P. Slijper, Ruud W. Selles, Robbert M. Wouters

**Affiliations:** 1 Department of Plastic, Reconstructive and Hand Surgery, Erasmus MC, University Medical Center Rotterdam, Rotterdam, The Netherlands; 2 Department of Rehabilitation Medicine, Erasmus MC, University Medical Center Rotterdam, Rotterdam, The Netherlands; 3 Center for Hand Therapy, Xpert Handtherapie, Eindhoven, The Netherlands; 4 Hand and Wrist Center, Xpert Clinics, Eindhoven, The Netherlands; 5 Department of Plastic, Reconstructive and Hand Surgery, University Medical Center Utrecht, Utrecht, The Netherlands

## Abstract

**Background:**

Although nonsurgical treatment of thumb carpometacarpal (CMC-1) osteoarthritis (OA) provides short-term improvement, the durability of these effects beyond 1 year is unknown. In this study, we investigated patient-reported pain and limitations in activities of daily living (ADL) at >5 years following nonsurgical treatment (i.e., exercise therapy and use of an orthosis) for CMC-1 OA. We hypothesized that pain and limitations in ADL would not worsen after 12 months. Secondary outcomes were satisfaction with treatment results and health-related quality of life at >5 years of follow-up and the rate of conversion to surgery.

**Methods:**

This was a multicenter, prospective cohort study using 2 overlapping samples. The change in the Michigan Hand Outcomes Questionnaire (MHQ) subscales of pain and ADL between 12 months and >5 years was the primary outcome as measured in the first sample (n = 170), which consisted of patients who did not undergo conversion to surgery. Additional measurement time points included baseline and 3 months. We evaluated conversion to surgery in a second sample, which included all patients who responded to the invitation for this follow-up study (n = 217).

**Results:**

At a median follow-up of 6.6 years (range, 5.1 to 8.7 years), the score on the MHQ pain subscale did not differ significantly from that at 12 months. The score on the MHQ ADL improved by 4.4 points (95% confidence interval [CI],1.5 to 7.2) compared with 12 months, but this was not clinically relevant. At >5 years, 5% of the patients rated their satisfaction as “poor,” 14% as “moderate,” 26% as “fair,” 39% as “good,” and 16% as “excellent.” The median EuroQol-5 Dimensions-5 Levels (EQ-5D-5L) index score was 0.852 (range, 0.135 to 1). The rate of conversion to surgery was 22% (95% CI,16.4% to 27.7%) at a median follow-up of 7 years (range, 5.5 to 9.0 years).

**Conclusions:**

We found positive outcomes at >5 years of follow-up for nonsurgical treatment of CMC-1 OA, with no worsening of pain or of limitations in ADL after 12 months. Our findings support nonsurgical treatment as the first treatment choice and suggest that treatment effects are sustainable.

**Level of evidence:**

Therapeutic Level II. See Instructions for Authors for a complete description of levels of evidence.

Osteoarthritis (OA) of the thumb carpometacarpal joint (CMC-1) is a common disorder with a symptomatic prevalence of 7% for females and 2% for males >50 years of age^1-3^. Because of the aging population, the number of patients with CMC-1 OA is expected to increase^3,4^. CMC-1 OA often results in pain, limitations in activities of daily living (ADL), reduced quality of life, thenar muscle wasting, and/or thumb deformity^1,2^.

Most guidelines for the management of CMC-1 OA advise starting with nonsurgical treatment, such as the use of an orthosis, corticosteroid injections, analgesics, exercise therapy, or a combination of these treatments^5-7^. Surgery can be considered when nonsurgical treatment fails to sufficiently relieve symptoms^7-9^.

Although short-term effectiveness has been demonstrated for the combination of exercise therapy and orthosis use^7,10-12^, the long-term effects are unknown^7,11,13,14^. Exercise therapy can optimize thumb positioning and improve use in daily life, with a known treatment effect up to 1 year^11^. Because of the chronic nature of CMC-1 OA, knowledge of the long-term outcomes is needed to provide patients and clinicians with greater insight into the durability of nonoperative management. As treatment focuses on stable thumb positioning and improved use in daily life, we hypothesized that the treatment effects of exercise therapy plus use of an orthosis remain stable from 1 year up to at least 5 years and that this treatment thus offers a sustainable solution. Furthermore, while guidelines advise initial nonsurgical treatment to potentially avoid surgery, the actual long-term conversion to surgery has been poorly described. Few studies have reported on conversion to surgery and typically have had a follow-up of approximately 2 years^11,15,16^. Avoiding surgery is important if possible because of the lengthy associated rehabilitation, costs, and outcome variation; the results are not always to the patients’ satisfaction^17^.

In the current study, we investigated pain and limitations in ADL following nonsurgical treatment (consisting of exercise therapy and use of an orthosis) for CMC-1 OA at >5 years and tested the hypothesis that these outcomes would not deteriorate after 12 months in patients who did not convert to surgery. Secondary and exploratory outcomes included satisfaction with treatment results and health-related quality of life at >5 years, and the rate of conversion to surgery following nonsurgical treatment of CMC-1 OA.

## Materials and Methods

### Study Design and Setting

This was a multicenter, prospective cohort study that followed the guidelines of the STROBE (Strengthening the Reporting of Observational Studies in Epidemiology) statement^18^. All patients provided informed consent, and the institutional review board approved the study.

Data were collected during the study period at Xpert Clinics, comprising 8 specialized hand clinics with 18 hand surgeons in The Netherlands. Outcomes up to 12 months were routinely measured using GemsTracker (GEneric Medical Survey Tracker) software (Erasmus and Equipe Zorgbedrijven); details of our data collection were previously published^10,11,19^. On the basis of the combination of the patient’s diagnosis and selected treatment, each patient was assigned a “measurement track,” which includes predefined measurements at predefined time points^20^. For the current study, patients were invited to complete prospectively distributed questionnaires >5 years after treatment onset.

### Participants

All patients were diagnosed with CMC-1 OA by hand surgeons on the basis of symptoms and physical examination and referred for hand therapy between January 2011 and October 2015. Radiographs are usually made but not routinely recorded. We invited patients to participate in this follow-up study if they had completed the visual analog scale (VAS) for pain at rest, pain during activities, and function, and the Michigan Hand Outcomes Questionnaire (MHQ) at baseline and 3 months as part of their routine outcome assessments.

We used 2 overlapping samples in this study, whereby sample 1 was a subset of sample 2. In sample 1, we studied pain and limitations in ADL of patients who did not have surgery. We used sample 2 to evaluate conversion to surgery. For both samples, we included adult patients with complete sociodemographic data at baseline, scores on the MHQ at baseline and 3 months, and data from our follow-up questionnaires at >5 years. Exclusion criteria for both samples were CMC-1 surgery before nonsurgical treatment, posttraumatic CMC-1 OA, a simultaneous intervention for another hand or wrist comorbidity, and a corticosteroid injection <6 weeks before treatment. For sample 1, an additional criterion was that the patient did not undergo conversion to surgery. Information regarding these criteria was extracted from patient records and our data collection system.

### Intervention

Trained hand therapists carried out the treatment at the 8 specialized hand clinics. Treatment was based on the local and Dutch treatment guidelines, generally consisting of the use of a custom-made or prefabricated orthosis, weekly 25-minute therapy sessions including exercises and education to achieve a more stable opposition position, and daily exercises^5^. A detailed description of the treatment is presented in Appendix 1. Treatment frequency and duration differed by patient and were determined by the therapist in conjunction with the patient, inherent to the study’s observational nature.

### Measurements

Baseline demographics, including sex, age, type of work, hand dominance, affected hand, and symptom duration, were retrieved from our database.

The primary outcome was the change in pain and limitations in ADL between 12 months and >5 years. Pain and limitations in ADL were routinely measured with the MHQ subscales of pain and ADL at baseline, 3 months, and 1 year. For this study, additional MHQ data were collected prospectively at >5 years. The MHQ has high internal consistency, validity, and acceptable reliability^21^. Scores on the MHQ subscales of overall hand function, work performance, aesthetics, and satisfaction with hand function were secondary outcomes^21,22^. MHQ scores range from 0 to 100; higher scores indicate better performance for all subscales except pain. We reversed the pain subscale to ease interpretation, and thus higher scores indicate better performance for all subscales. The MHQ pain and ADL subscales have a minimal important change (MIC) of 8 and 6, respectively, for nonsurgically treated CMC-1 OA^23^.

In addition to the MHQ, other questionnaires were distributed at the >5-year time point. We used the valid and reliable Satisfaction with Treatment Result Questionnaire, which evaluates satisfaction with treatment results on a 5-point Likert scale (poor, moderate, fair, good, and excellent) and asks whether the patient would undergo the treatment again under similar circumstances^24^.

The EuroQol-5 Dimensions-5 Levels (EQ-5D-5L) was used to measure health-related quality of life (HRQoL) at >5 years. Scores on the EQ-5D index are anchored at 1 (full health) and 0 (a state as bad as death; lower values indicate even worse health states)^25,26^. The EQ-5D-5L had good reliability, validity, and moderate responsiveness in patients with upper-extremity disorders^27^.

Data on conversion to surgery within our clinics were extracted from patient records and our database of all patients in sample 2. Additionally, we used a survey to ask patients at >5 years whether they had undergone surgery for their CMC-1 OA.

### Statistical Analysis

A sample size analysis based on our 4 repeated measures, a conventional effect size of 0.25^28^, alpha = 0.025 (based on a Bonferroni correction for the 2 primary outcomes of pain and ADL), and a power of 0.80 indicated that 49 participants were required. This was well below the achieved sample of 170.

We used linear mixed models with the MHQ subscales of pain and ADL as dependent variables and the time points as a fixed factor for sample 1. We also performed the same analyses for the other MHQ subscales. Assumptions were checked using residual plots and normal probability plots.

We performed a nonresponder analysis to investigate whether patients with missing values systematically differed from those without missing values, by comparing demographics and primary outcomes at baseline and 3 months between the participants who completed the MHQ at >5 years (defined as responders) and the participants who did not (defined as nonresponders), using t tests and chi-square tests. We performed a similar nonresponder analysis for missing data at 12 months. Additionally, we performed a Little’s test, which assesses the null hypothesis that the data are missing completely at random^29,30^.

We report descriptive statistics for HRQoL and satisfaction with treatment in sample 1 at >5 years. We report the rate of conversion to surgery and used a Kaplan-Meier survival curve to display the time, in months, at which patients in sample 2 decided to undergo conversion to surgery. All analyses were performed using R (version 4.0.3; R Foundation for Statistical Computing).

### Source of Funding

No external funding was received for this study.

## Results

**Fig. 1:**
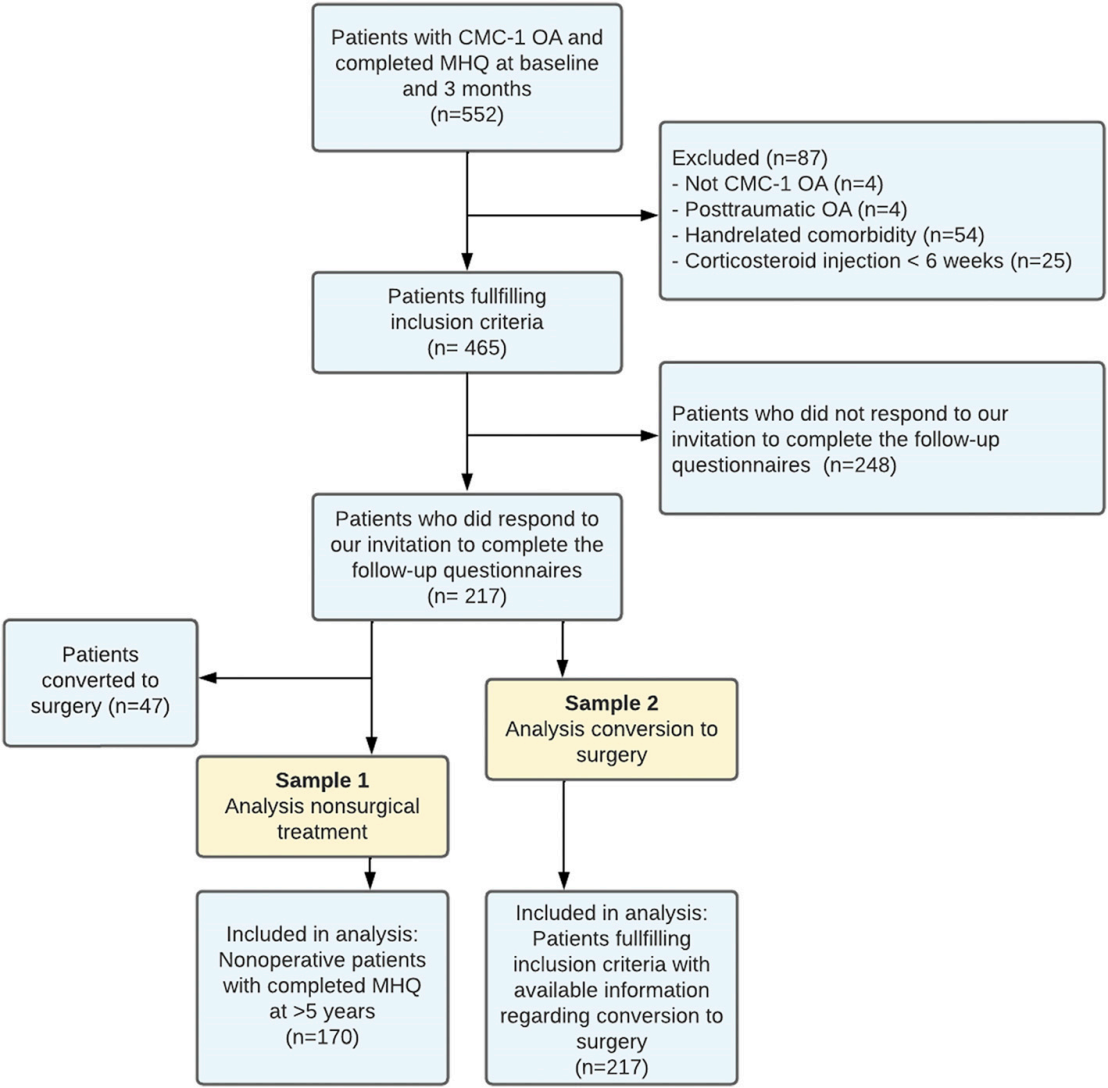
Flowchart for the study. CMC-1 = thumb carpometacarpal joint, OA = osteoarthritis, and MHQ = Michigan Hand Outcomes Questionnaire. Note that sample 1 is a subset of sample 2.

We invited 552 patients to participate in this study on the basis of their earlier assigned measurement track. We excluded 87 patients without CMC-1 OA, with traumatic CMC-1 OA, hand-related comorbidities, or a corticosteroid injection <6 weeks before treatment initiation (Fig. 1). After applying the additional exclusion criterion (no surgery) for sample 1, 170 of the responders were included in that sample, of whom 134 also had MHQ outcomes at 12 months. We included all 217 responders in the second sample. The baseline characteristics of both samples are given in Table I.

**TABLE I:** Baseline Patient Characteristics

Variable	Sample 1 (N = 170)	Sample 2 (N = 217)
Age* *(yr)*	59 ± 8	59 ± 8
No. (%) female	129 (76)	172 (79)
Symptom duration† *(mo)*	12 [6-36]	12 [6-36]
Dominant hand *(no. [%])*		
Left	14 (8)	19 (9)
Right	147 (87)	189 (87)
Both	9 (5)	9 (4)
Treated hand *(no. [%])*		
Left	96 (57)	114 (53)
Right	74 (44)	103 (48)
Type of work *(no. [%])*		
Unemployed	70 (41)	83 (38)
Light physical work	37 (22)	52 (24)
Moderate physical work	41 (24)	54 (25)
Heavy physical work	22 (13)	28 (13)
MHQ score*		
Total	61 ± 14	59 ± 14
ADL	69 ± 19	66 ± 20
Pain	49 ± 16	46 ± 16
Function	58 ± 15	57 ± 15
Aesthetics	84 ± 20	83 ± 20
Satisfaction	44 ± 22	42 ± 22
Work performance	63 ± 25	61 ± 25

* The values are given as the mean and standard deviation. MHQ = Michigan Hand Outcomes Questionnaire, and ADL = activities of daily living.

† The values are given as the median, with the interquartile range in square brackets.

The 2 nonresponder analyses indicated that at >5 years, only 1 of 21, and at 12 months, 2 of 21 variables differed between responders and nonresponders (see Appendix Supplementary Tables 1 and 2). The nonsignificant results of Little’s test (p = 0.175) further suggest that the data were missing completely at random^29,30^.

### Primary and Secondary Outcomes

**Fig. 2:**
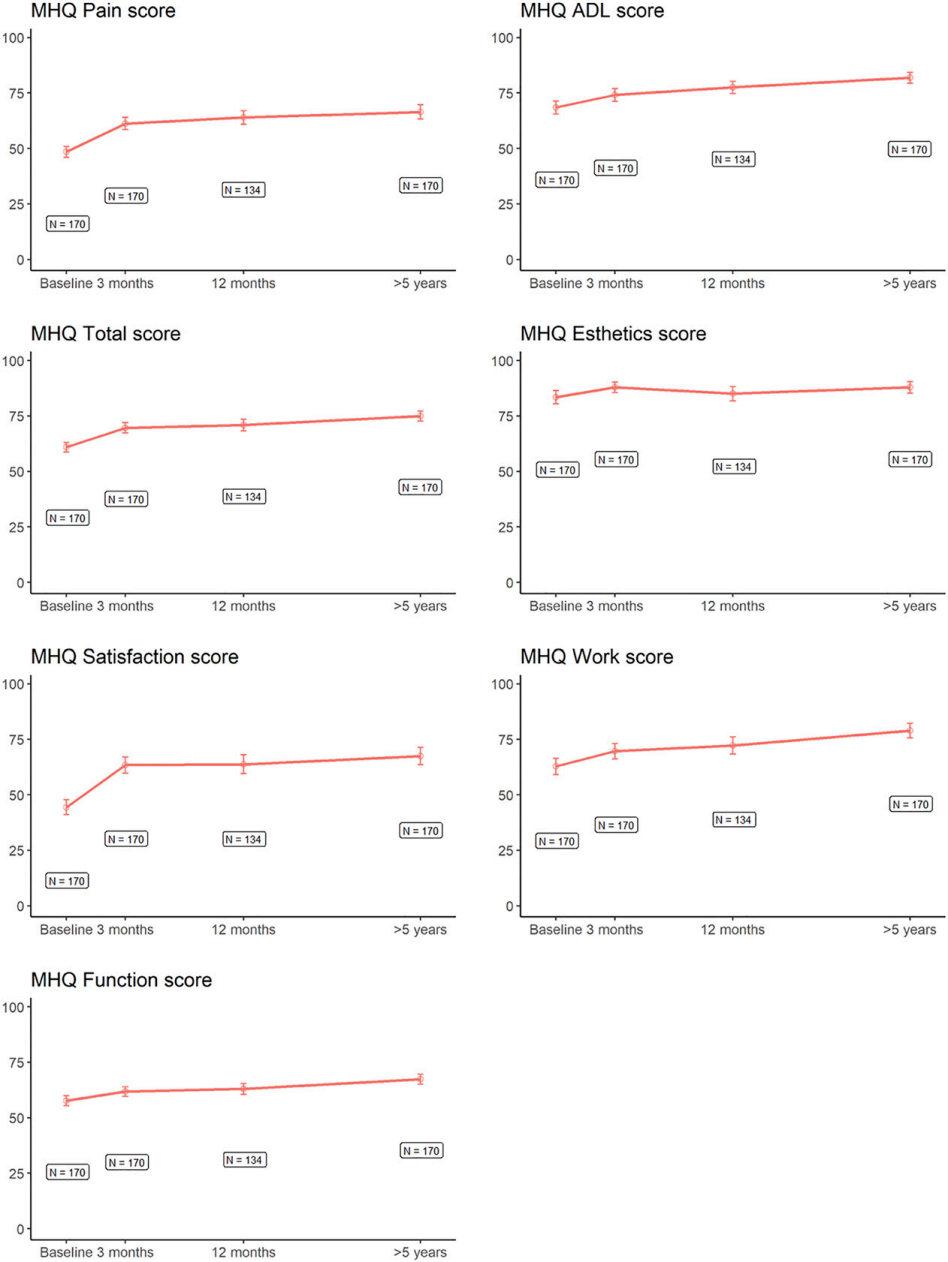
Mean scores on the Michigan Hand Outcomes Questionnaire (MHQ) subscales over time. Most improvement was seen in the first 3 months. The error bars show the 95% confidence interval.

The median follow-up duration was 6.6 years (range, 5.1 to 8.7 years). Fig. 2 demonstrates scores on the MHQ over time among the nonsurgical patients in sample 1. Scores on the MHQ pain subscale did not change between 12 months and >5 years. Scores on the MHQ ADL improved by 4.4 points (95% confidence interval [CI], 1.5 to 7.2), which was significant (p ≤ 0.0137) but not clinically relevant (Table II). The MHQ total score and scores on the subscales of overall hand function and work performance also improved significantly between 12 months and >5 years, and this improvement was clinically relevant. We observed no differences in scores on the other subscales (aesthetics and satisfaction with hand function) (Table II).

**TABLE II:** Changes in the MHQ Total Score and Subscales Over Time in Sample 1*

	Change					
MHQ Score	Baseline to 3 Mo	Baseline to 12 Mo	Baseline to 5 Yr	3 Mo to 12 Mo	3 Mo to >5 Yr	12 Mo to >5 Yr
Total	8.8†	10.0†	14.1†	1.2 (NS)	5.3†	4.2‡
ADL	5.6‡	9.0†	13.4†	3.4 (NS)	7.8†	4.4§
Pain	12.7†	15.2†	17.9†	2.5 (NS)	5.2‡	2.7 (NS)
Function	4.1‡	5.1‡	9.7†	1.0 (NS)	5.7†	4.6‡
Aesthetics	4.5§	1.8 (NS)	4.4 (NS)	–2.6 (NS)	–0.1 (NS)	2.5 (NS)
Satisfaction	18.9†	18.7†	23.0†	–0.2 (NS)	4.1 (NS)	4.3 (NS)
Work performance	6.8‡	9.8†	16.1†	3.0 (NS)	9.3†	6.3§

* Scores range from 0 to 100; higher scores indicate better function and less pain. Significance testing was performed using linear mixed-model analysis. The changes represent the marginal mean difference between the given time points as estimated by the model. MHQ = Michigan Hand Outcomes Questionnaire, and NS = not significant.

† Significant at p < 0.0001.

‡ Significant at p < 0.010.

§ Significant at p < 0.025.

**Fig. 3:**
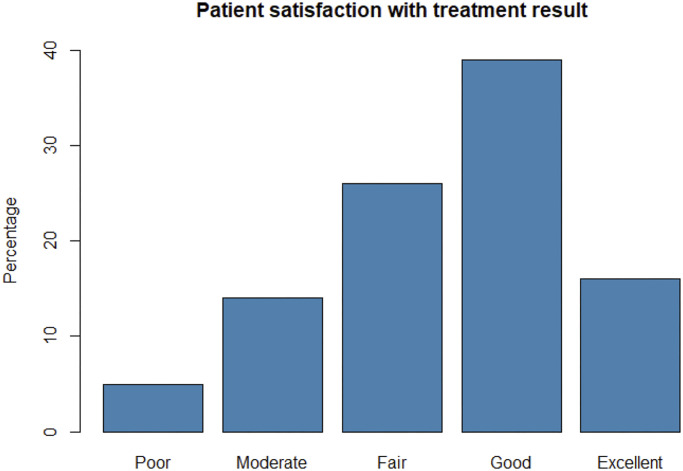
Patient satisfaction with the treatment result (n = 163), expressed in percentages.

One hundred and sixty-three participants in sample 1 completed the Satisfaction with Treatment Result Questionnaire at >5 years. Five percent of the respondents rated their satisfaction with the results as “poor,” 14% as “moderate,” 26% as “fair,” 39% as “good,” and 16% as “excellent” (Fig. 3). Seventy-one percent of the participants were willing to undergo the treatment again under similar circumstances. The median EQ-5D-5L index score was 0.852 (range, 0.135 to 1).

**Fig. 4:**
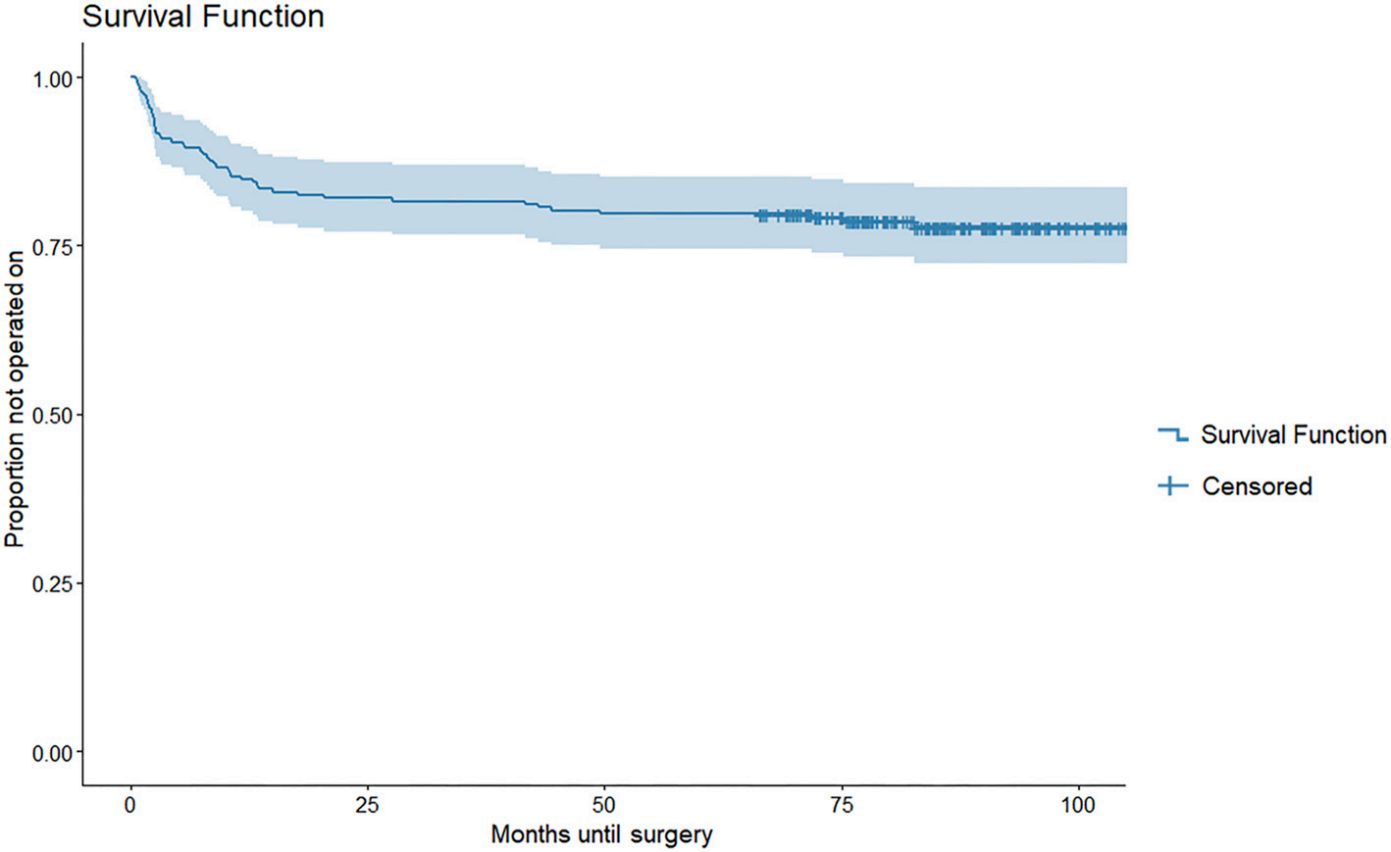
Survival curve. The blue line indicates the proportion of patients who did not undergo conversion to surgery and the time, in months, before deciding to convert to surgery. At a median follow-up of 7.0 years, 22% had decided to convert to surgery. The median time to decide to convert to surgery was 7.4 months after treatment initiation. The light blue shading indicates the 95% confidence interval. Censoring means that the patient had not converted to surgery at the time the study was conducted.

Figure 4 shows the survival curve for conversion to surgery, demonstrating that at a median follow-up of 7.0 years (range, 5.5 to 9.0 years), 47 (22%) of the 217 participants (95% CI, 16.4% to 27.7%) had converted to surgery. Of those patients, the majority (70%) converted to surgery within the first year after treatment. The median time at which patients decided to convert to surgery was 7.4 months (range, 0.7 to 82.7 months) after treatment initiation.

## Discussion

We found no clinically relevant change in pain and limitations in ADL between 12 months and >5 years following nonsurgical treatment for CMC-1 OA. These findings apply to patients with CMC-1 OA who did not convert to surgery during the follow-up interval. Our findings suggest that the improvement after nonsurgical treatment as measured in the first 12 months after treatment is sustainable. Secondary outcomes at >5 years of follow-up indicated relatively high satisfaction with treatment results, and only 22% had converted to surgery at a median follow-up of 7.0 years.

The long-term effect of the nonsurgical treatment of CMC-1 OA has not been well described. Although, in the current study, most improvements occurred in the active treatment period (i.e., the first 3 months), there were additional improvements seen at 12 months and even at >5 years in patients who did not convert to surgery. As we hypothesized, there was no deterioration in terms of pain or ADL limitations, as measured with the MHQ. A possible explanation for the lasting improvements could be that, apart from strengthening the thenar muscles, exercise therapy is aimed at using a new and more stable position of the thumb, thereby reducing joint loading and inflammation. Thus, patients learn how to prevent pain by using this position and to cope with their OA.

The HRQoL was assessed as a standalone cross-sectional outcome at >5 years. Since HRQoL is an underreported part of outcomes in this patient population, this outcome could be valuable for future research^31,32^. A study by Lane et al. reported EQ-5D index scores of 0.85 and 0.8 at 1 year postoperatively among patients who had surgery for CMC-1 OA, which are similar to, or slightly lower than, the median score in the present study^33^.

Our finding that only 22% converted to surgery is comparable with rates in other studies with shorter follow-up durations. Tsehaie et al. found a conversion rate of 15% in a study with a mean follow-up of 2.2 years and including a cohort with some of the same patients as in the present study^11^. Gravås et al. reported that 24% of the patients who received occupational therapy in their randomized controlled trial had undergone surgery during a follow-up of 2 years^15^. In another study, Schloemann et al. reported a conversion-to-surgery rate of 9% with a median follow-up of 1.5 years^16^. These differences in conversion rates may be due to differences in populations or treatment protocols. Nevertheless, the findings that the conversion rate at approximately 2 years is comparable with the conversion rate at >5 years indicates that few patients undergo surgery once the positive treatment effect has been achieved, which further supports nonsurgical treatment as a first treatment choice.

### Study Limitations

A limitation of this study is the missing data, which are inherent to the observational nature of this study. However, our nonresponder analyses suggested that the data were missing at random; there was a slight age difference between the responders and nonresponders, which seems clinically irrelevant, and no differences in the MHQ scores between the responders and nonresponders. The nonsignificant results of Little’s test further suggests that the missing data were missing completely at random. Therefore, we are confident that these missing data did not influence our primary findings.

Related to the observational design and associated missing data, our ability to estimate the true rate of conversion to surgery may have been limited, as the patients for whom we had data on conversion to surgery may not have been fully representative of the target population. This means that some patients may have sought care elsewhere without our knowledge (i.e., the conversion rate could actually be higher) or that patients without symptoms who had not undergone surgery did not respond to our survey (i.e., the conversion rate could actually be lower). However, all patients who did not undergo surgery according to our patient records also responded to the survey regarding additional treatments by indicating that they had not undergone surgery, suggesting that we did not underestimate the conversion rate. Still, this is a limitation, and future studies should address this in their design. Furthermore, insights into factors associated with conversion to surgery may enable faster, well-founded decision-making. Previous studies found that pain improvement, prior surgical experience, surgeons’ attitudes toward CMC-1 OA, previous nonpharmacological treatment, and higher motivation for surgery are influential^11,15,16^. Predicting conversion to surgery may be improved by also considering the psychological characteristics of patients. A previous study found that patients scheduled for surgical treatment have a worse psychological profile than their nonsurgical counterparts^34^. Because of the relatively low number of patients who converted to surgery, we could not assess that in our study.

Another limitation is that our patient-reported outcomes only represent patients who did not convert to surgery. This may have introduced selection bias because patients who eventually received surgery most likely had worse outcomes. Therefore, these outcomes only apply to patients who had not undergone surgery by the final follow-up of >5 years. However, since most surgeries occurred within the first year after treatment onset, the findings between 12 months and >5 years seem unaffected by this risk of bias.

Finally, a limitation of our observational design is that we cannot prove causality. However, the results have a higher ecological validity, as our findings reflect daily practice. Similarly, although therapists are trained the same, there may have been variations in the treatment they provided, and treatment frequency. This possible variation is important to acknowledge because it has previously been shown that exercises have a relatively large treatment effect compared with that of orthosis use alone^10^. However, this variation is also a strength as it reflects daily clinical practice. To prove causality, a no-treatment arm would have to be included as a control group, since the natural course of CMC-1 OA may be somewhat self-limited regarding pain.

### Conclusions

We found positive outcomes at >5 years of follow-up after the nonsurgical treatment of CMC-1 OA, with no worsening of pain or limitations in ADL after 12 months, and with only 22% of the participants having converted to surgery at a median follow-up of 7 years. Our findings support nonsurgical treatment as the first treatment choice and indicate that treatment effects are sustainable.

## Appendix

Supporting material provided by the authors is posted with the online version of this article as a data supplement at jbjs.org (http://links.lww.com/JBJS/H689).

## References

[1] Bijlsma JWJ Berenbaum F Lafeber FPJG. Osteoarthritis: an update with relevance for clinical practice. Lancet. 2011 Jun 18;377(9783):2115-26. 21684382 10.1016/S0140-6736(11)60243-2

[2] Haugen IK Englund M Aliabadi P Niu J Clancy M Kvien TK Felson DT. Prevalence, incidence and progression of hand osteoarthritis in the general population: the Framingham Osteoarthritis Study. Ann Rheum Dis. 2011 Sep;70(9):1581-6. 21622766 10.1136/ard.2011.150078 PMC3867970

[3] van der Oest MJW Duraku LS Andrinopoulou ER Wouters RM Bierma-Zeinstra SMA Selles RW Zuidam JM. The prevalence of radiographic thumb base osteoarthritis: a meta-analysis. Osteoarthritis Cartilage. 2021 Jun;29(6):785-792. 33744429 10.1016/j.joca.2021.03.004

[4] Centraal Bureau voor de Statistiek. Population; key figures, 1950-2022. 2023. Accessed 2023 Jul 27. https://www.cbs.nl/nl-nl/cijfers/detail/37296ned.

[5] Nederlandse Vereniging voor Handchirurgie. Conservatieve en Chirurgische Behandeling van Primaire Artrose van de Duimbasis. 2014. Accessed 2023 Jul 27. https://www.nvpc.nl/uploads/stand/150416DOC-MB-Definitieve_richtlijn_Conservatieve_en_Chirurgische_behandeling_duimbasisartrose_28-10-2014_aangenomen_ALV_14_april_2015149.pdf.

[6] Kolasinski SL Neogi T Hochberg MC Oatis C Guyatt G Block J Callahan L Copenhaver C Dodge C Felson D Gellar K Harvey WF Hawker G Herzig E Kwoh CK Nelson AE Samuels J Scanzello C White D Wise B Altman RD DiRenzo D Fontanarosa J Giradi G Ishimori M Misra D Shah AA Shmagel AK Thoma LM Turgunbaev M Turner AS Reston J. 2019 American College of Rheumatology/Arthritis Foundation Guideline for the Management of Osteoarthritis of the Hand, Hip, and Knee. Arthritis Care Res (Hoboken). 2020 Feb;72(2):149-62. 31908149 10.1002/acr.24131 PMC11488261

[7] Bertozzi L Valdes K Vanti C Negrini S Pillastrini P Villafañe JH. Investigation of the effect of conservative interventions in thumb carpometacarpal osteoarthritis: systematic review and meta-analysis. Disabil Rehabil. 2015;37(22):2025-43. 25559974 10.3109/09638288.2014.996299

[8] Buhler M Chapple CM Stebbings S Sangelaji B Baxter GD. Effectiveness of splinting for pain and function in people with thumb carpometacarpal osteoarthritis: a systematic review with meta-analysis. Osteoarthritis Cartilage. 2019 Apr;27(4):547-59. 30317000 10.1016/j.joca.2018.09.012

[9] Kroon FPB Rubio R Schoones JW Kloppenburg M. Intra-Articular Therapies in the Treatment of Hand Osteoarthritis: A Systematic Literature Review. Drugs Aging. 2016 Feb;33(2):119-33. 26650235 10.1007/s40266-015-0330-5 PMC4756050

[10] Wouters RM Tsehaie J Slijper HP Hovius SER Feitz R Selles RW; Hand-Wrist Study Group. Exercise Therapy in Addition to an Orthosis Reduces Pain More Than an Orthosis Alone in Patients With Thumb Base Osteoarthritis: A Propensity Score Matching Study. Arch Phys Med Rehabil. 2019 Jun;100(6):1050-60. 30543802 10.1016/j.apmr.2018.11.010

[11] Tsehaie J Spekreijse KR Wouters RM Slijper HP Feitz R Hovius SER Selles RW. Outcome of a Hand Orthosis and Hand Therapy for Carpometacarpal Osteoarthritis in Daily Practice: A Prospective Cohort Study. J Hand Surg Am. 2018 Nov;43(11):1000-1009.e1. 29776723 10.1016/j.jhsa.2018.04.014

[12] Villafañe JH Valdes K Pedersini P Berjano P. Thumb carpometacarpal osteoarthritis: A musculoskeletal physiotherapy perspective. J Bodyw Mov Ther. 2019 Oct;23(4):908-12. 31733781 10.1016/j.jbmt.2019.02.018

[13] O’Brien VH Giveans MR. Effects of a dynamic stability approach in conservative intervention of the carpometacarpal joint of the thumb: a retrospective study. J Hand Ther. 2013 Jan-Mar;26(1):44-51, quiz 52. 23177671 10.1016/j.jht.2012.10.005

[14] Deveza LA Robbins SR Duong V Bennell KL Vicenzino B Hodges PW Wajon A Jongs R Riordan EA Fu K Oo WM O’Connell RL Eyles JP Hunter DJ. Efficacy of a Combination of Conservative Therapies vs an Education Comparator on Clinical Outcomes in Thumb Base Osteoarthritis: A Randomized Clinical Trial. JAMA Intern Med. 2021 Apr 1;181(4):429-38. 33683300 10.1001/jamainternmed.2020.7101 PMC7941246

[15] Gravås EMH Østerås N Nossum R Eide REM Klokkeide Å Matre KH Olsen M Andreassen O Haugen IK Tveter AT Kjeken I. Does occupational therapy delay or reduce the proportion of patients that receives thumb carpometacarpal joint surgery? A multicentre randomised controlled trial. RMD Open. 2019 Nov 6;5(2):e001046. 31798953 10.1136/rmdopen-2019-001046 PMC6861078

[16] Schloemann D Hammert WC Liu S Bernstein DN Calfee RP. Risk Factors for Failed Nonsurgical Treatment Resulting in Surgery on Thumb Carpometacarpal Arthritis. J Hand Surg Am. 2021 Jun;46(6):471-477.e1. 33832788 10.1016/j.jhsa.2021.02.009

[17] Vermeulen GM Brink SM Slijper H Feitz R Moojen TM Hovius SER Selles RW. Trapeziometacarpal arthrodesis or trapeziectomy with ligament reconstruction in primary trapeziometacarpal osteoarthritis: a randomized controlled trial. J Bone Joint Surg Am. 2014 May 7;96(9):726-33. 24806009 10.2106/JBJS.L.01344

[18] von Elm E Altman DG Egger M Pocock SJ Gøtzsche PC Vandenbroucke JP; STROBE Initiative. The strengthening the reporting of observational studies in epidemiology (STROBE) statement: Guidelines for reporting observational studies. Int J Surg. 2014 Dec;12(12):1495-9. 25046131 10.1016/j.ijsu.2014.07.013

[19] GemsTracker. Home. Accessed 2023 Jul 27. https://gemstracker.org/.

[20] Selles RW Wouters RM Poelstra R van der Oest MJW Porsius JT Hovius SER Moojen TM van Kooij Y Pennehouat PY van Huis R Vermeulen GM Feitz R Slijper HP; Hand-Wrist Study Group. Routine Health Outcome Measurement: Development, Design, and Implementation of the Hand and Wrist Cohort. Plast Reconstr Surg. 2020 Aug;146(2):343-54. 32740587 10.1097/PRS.0000000000007008

[21] Poole JL. Measures of hand function: Arthritis Hand Function Test (AHFT), Australian Canadian Osteoarthritis Hand Index (AUSCAN), Cochin Hand Function Scale, Functional Index for Hand Osteoarthritis (FIHOA), Grip Ability Test (GAT), Jebsen Hand Function Test (JHFT), and Michigan Hand Outcomes Questionnaire (MHQ). Arthritis Care Res (Hoboken). 2011 Nov;63(Suppl 11):S189-99. 22588744 10.1002/acr.20631

[22] Kroon FPB Boersma A Boonen A van Beest S Damman W van der Heijde D Rosendaal FR Kloppenburg M. Performance of the Michigan Hand Outcomes Questionnaire in hand osteoarthritis. Osteoarthritis Cartilage. 2018 Dec;26(12):1627-35. 30099114 10.1016/j.joca.2018.07.018

[23] Hoogendam L Koopman JE van Kooij YE Feitz R Hundepool CA Zhou C Slijper HP Selles RW Wouters RM; The Hand-Wrist Study Group. What Are the Minimally Important Changes of Four Commonly Used Patient-reported Outcome Measures for 36 Hand and Wrist Condition-Treatment Combinations? Clin Orthop Relat Res. 2022 Jun 1;480(6):1152-66. 34962496 10.1097/CORR.0000000000002094 PMC9263468

[24] De Ridder WA van Kooij YE Vermeulen GM Slijper HP Selles RW Wouters RM. Test-retest Reliability and Construct Validity of the Satisfaction with Treatment Result Questionnaire in Patients with Hand and Wrist Conditions: A Prospective Study. Clin Orthop Relat Res. 2021 Sep 1;479(9):2022-2032. 34014631 10.1097/CORR.0000000000001794 PMC8373545

[25] M Versteegh M M Vermeulen K M A A Evers S de Wit GA Prenger R A Stolk E. Dutch Tariff for the Five-Level Version of EQ-5D. Value Health. 2016 Jun;19(4):343-52. 27325326 10.1016/j.jval.2016.01.003

[26] Buchholz I Janssen MF Kohlmann T Feng YS. A Systematic Review of Studies Comparing the Measurement Properties of the Three-Level and Five-Level Versions of the EQ-5D. Pharmacoeconomics. 2018 Jun;36(6):645-61. 29572719 10.1007/s40273-018-0642-5 PMC5954044

[27] Grobet C Marks M Tecklenburg L Audigé L. Application and measurement properties of EQ-5D to measure quality of life in patients with upper extremity orthopaedic disorders: a systematic literature review. Arch Orthop Trauma Surg. 2018 Jul;138(7):953-61. 29654354 10.1007/s00402-018-2933-x

[28] Cohen J. Statistical power analysis for the behavioral sciences. Lawrence Erlbaum Associates; 1988.

[29] de Groot JAH Janssen KJM Zwinderman AH Bossuyt PMM Reitsma JB Moons KGM. Correcting for partial verification bias: a comparison of methods. Ann Epidemiol. 2011 Feb;21(2):139-48. 21109454 10.1016/j.annepidem.2010.10.004

[30] Little RJA. A test of missing completely at random for multivariate data with missing values. J Am Stat Assoc. 1988;83(404):1198-202.

[31] Michon M Maheu E Berenbaum F. Assessing health-related quality of life in hand osteoarthritis: a literature review. Ann Rheum Dis. 2011 Jun;70(6):921-8. 21398333 10.1136/ard.2010.131151

[32] Østerås N Kjeken I Smedslund G Moe RH Slatkowsky-Christensen B Uhlig T Hagen KB. Exercise for hand osteoarthritis: A Cochrane systematic review. J Rheumatol. 2017 Dec;44(12):1850-8. 29032354 10.3899/jrheum.170424

[33] Lane JCE Rodrigues JN Furniss D Burn E Poulter R Gardiner MD. Basal thumb osteoarthritis surgery improves health state utility irrespective of technique: a study of UK Hand Registry data. J Hand Surg Eur Vol. 2020 Jun;45(5):436-42. 32162998 10.1177/1753193420909753 PMC7232779

[34] Wouters RM Vranceanu AM Slijper HP Vermeulen GM van der Oest MJW Selles RW Porsius JT; Hand-Wrist Study Group. Patients With Thumb-base Osteoarthritis Scheduled for Surgery Have More Symptoms, Worse Psychological Profile, and Higher Expectations Than Nonsurgical Counterparts: A Large Cohort Analysis. Clin Orthop Relat Res. 2019 Dec;477(12):2735-46. 31764344 10.1097/CORR.0000000000000897 PMC6907312

